# Prediction of mortality of APACHE-II and APACHE-III scores in patients admitted to the intensive care unit (ICU) for intoxication

**DOI:** 10.1186/2197-425X-3-S1-A342

**Published:** 2015-10-01

**Authors:** E Banderas-Bravo, MD Arias-Verdú, I Macias-Guarasa, E Aguilar-Alonso, E Castillo-Lorente, R Rivera-Fernández, G Quesada- Garcia

**Affiliations:** Intensive Care Unit, Hospital Regional Universitario Carlos Haya, Málaga, Spain; Intensive Care Unit, Hospital Infanta Margarita Cabra, Córdoba, Spain; Intensive Care Unit, Hospital Neurotraumatologico, Jaen, Spain

## Objective

To study the characteristics and mortality in intoxicated patients admitted to the ICU, and prediction of mortality for usual prognostic indexes.

## Methods

A multicenter study conducted from 2008 to 2013 in three Spanish hospitals. We collected clinical and demographics data. The differences between observed-to-predicted mortality were analyzed whith the Hosmer-Lemeshow test. Discrimination was evaluated by área under the ROC curve. P < 0.005 was statistically significant.

## Results

We studied 119 patients. Mean age: 44.42 ± 13.85 years. Drug intoxication in 77.3% of patients, alcohol in 16.8% and caustic 9.2%. 78.3% was a suicide attempt. The mean Glasgow Coma Scale in 72.5% were < 8 points. 69.7% required mechanical ventilation. The mortality of patients hospitalized for caustic ingestion was 54.5% and 1.9% in the rest (p < 0.001).

The multivariate analysis showed that with SAPS-3 equal gravity (OR: 1.19 (1.02-1.39)) the mortality of patients who ingested caustics was higher (OR: 560.34 (11.64-26.973.83)) than the rest.

The mortality predicted by SAPS-3 (general equation) was 26.98% and the observed 6.7%, Hosmer-Lemeshow (H = 36.47) (p < 0.001). With the APACHE-II and APACHE-III scores no statistically significant differences were observed, being the predicted mortality for APACHE-II of 7.57% (Hosmer-Lemeshow Test: H = 6.96, no difference is) and for APACHE-III of 8.15% (Hosmer-Lemeshow Test: C = 3.51, not discrepancies).

## Conclusions

APACHE-II and APACHE-III scores may adequately predict the probability of death in intoxicated patients, in contradistinction to the SAPS-3 score that overestimates the mortality. The ICU admission by intoxication is rare. A high percentage of patients have decreased level of consciousness and required mechanical ventilation. The mortality o f patients hospitalized for caustic ingestion is higher to the rest.

